# Characterization of lung stem cell niches in a mouse model of bleomycin-induced fibrosis

**DOI:** 10.1186/scrt112

**Published:** 2012-05-29

**Authors:** Ena Ray Banerjee, William Reed Henderson

**Affiliations:** 1 Department of Medicine, Division of Allergy and Infectious Diseases, Center for Allergy and Inflammation, University of Washington, Room 254, 815 Mercer Street, Seattle, WA, 98195, USA; 2 Associate Professor, Dept of Zoology, University of Calcutta, 35, Ballygunge Circular Road, Kolkata- 700019, West Bengal, India; 3 Professor and Head, Department of Medicine, Division of Allergy and Infectious Diseases, Center for Allergy and Inflammation, University of Washington, Room 254, 815 Mercer Street, Seattle, WA, 98195, USA

## Abstract

**Introduction:**

In lung fibrosis, alveolar epithelium degenerates progressively. The goal of regenerative medicine is to aid repair and regeneration of the lost tissues in parenchyma and airways for which mobilization of tissue-resident endogenous or bone marrow-derived exogenous stem cells niches is a critical step. We used a lung injury model in mice to identify and characterize functional lung stem cells to clarify how stem cell niches counteract this degenerative process.

**Methods:**

*Short term assay (STA) *- Bleomycin-induced lung inflammation and fibrosis were assessed in a model of idiopathic pulmonary fibrosis in wild-type (WT), gp91phox-/- (NOX-/-), and gp91phoxMMP-12 double knockout (DKO) mice on C57Bl/6 background and Hoechst 33322 dye effluxing side population (SP) cells characterized. *Long term assay (LTA) *- In a bleomycin induced lung fibrosis model in C57Bl6 mice, the number of mature cells were quantified over 7, 14, and 21 days in bone marrow (BM), peripheral blood (PB), lung parenchyma (LP) and brochoalveolar lavage (BAL) fluid by FACS. BrdU pulse chase experiment (10 weeks) was used to identify label retaining cells (LRC). BrdU^+ ^and BrdU^- ^cells were characterized by hematopoietic (CD45^+^), pluripotency (TTF1^+^, Oct3/4^+^, SSEA-3^+^, SSEA-4^+^, Sca1^+^, Lin^-^, CD34^+^, CD31^+^), and lung lineage-specific (SPC^+^, AQP-5^+^, CC-10^+^) markers. Clonogenic potential of LRCs were measured by CFU-c assays.

**Results:**

*STA*- In lung, cellularity increased by 5-fold in WT and 6-fold in NOX-/- by d7. Lung epithelial markers were very low in expression in all SP flow sorted from lung of all three genotypes cultured *ex vivo*. (p < 0.01). Post-bleomycin, the SP in NOX-/- lung increased by 3.6-fold over WT where it increased by 20-fold over controls. Type I and II alveolar epithelial cells progressively diminished in all three genotypes by d21 post-bleomycin. D7 post-bleomycin, CD45+ cells in BALf in NOX-/- was 1.7-fold > WT, 57% of which were Mf that decreased by 67% in WT and 83% in NOX-/- by d21.*LTA*- Cellularity as a factor of time remained unchanged in BM, PB, LP and BAL fluid. BrdU^+ ^(LRC) were the putative stem cells. BrdU^+^CD45^+ ^cells increased by 0.7-fold and SPC^+^CC10^+ ^bronchoalveolar stem cells (BASC), decreased by ~40-fold post-bleomycin. BrdU^+^VEGF^+ ^cells decreased by 1.8-fold while BrdU^-^VEGF^+ ^cells increased 4.6-fold. Most BrdU^- ^cells were CD45^-^. BrdU^- ^BASCs remained unchanged post-bleomycin. CFU-c of the flow-sorted BrdU^+ ^cells remained similar in control and bleomycin-treated lungs.

**Conclusion:**

*STA*- Inflammation is a pre-requisite for fibrosis; SP cells, being the putative stem cells in the lungs, were increased (either by self renewal or by recruitment from the exogenous bone marrow pool) post-bleomycin in NOX-/- but not in DKO indicating the necessity of cross-talk between gp91phox and MMP-12 in this process; *ex vivo *cultured SP progressively lose pluripotent markers, notably BASC (SPC+CC10+) - significance is unknown. *LTA***- **The increase in the hematopoietic progenitor pool in lung indicated that exogenous progenitors from circulation contribute to lung regeneration. Most non-stem cells were non-hematopoietic in origin indicating that despite tissue turnover, BASCs are drastically depleted possibly necessitating recruitment of progenitors from the hematopoietic pool. Loss of VEGF^+ ^LRC may indicate a signal for progenitor mobilization from niches. BrdU^- ^BASC population may be a small quiescent population that remains as a reserve for more severe lung injury. Increase in VEGF^+ ^non-LRC may indicate a checkpoint to counterbalance the mobilization of VEGF^+ ^cells from the stem cell niche.

## Introduction

Tissue injury and repair are ongoing processes in the lung and result from acute and chronic exposure to environmental insults. There are a myriad of effectors of lung injury, including infectious agents, particulate and chemical pollutants, radiation, and host defense mechanisms gone awry. Many of these processes are ablative in nature and require repair mechanisms that regenerate mature lung tissue through cell proliferation and differentiation.

Fundamental to understanding mechanisms of repair are identifying and characterizing the cells that are potentially capable of repopulating the injured tissue. Currently, efforts are being made to identify 1) which cell(s) repopulates regions of injured lung; 2) what their source is (endogenous or resident cells vs. exogenous or recruited cells) and 3) whether they are pluripotent stem cells capable of self renewal or transient amplifying cells that are multipoint but more lineage-committed. In the lung, multiple cell populations contribute to lung repair [1]. Most, like the basal cells of the tracheal epithelium, alveolar type II cells, bone marrow-derived stem cells, and residential stem cells that potentially serve the vascular compartment appear to be anatomically localized [2,3]. Others, like the side population (SP) cells, have not yet been localized to a single lung compartment. The molecular phenotype of hematopoietic stem cells (HSC) has been extensively characterized and is defined as a population of cells that are CD45^+^, Sca-1^+^, c-kit^+^, and Lin^-^. HSC are further characterized by their ability to rapidly efflux the DNA dye Hoechst 33342 [4,5].

The existence of putative lung tissue stem cells has only been suggested relatively recently through the use of rodent injury models in which abundant progenitor cells are depleted through either chemical or physical means [6-9]. At least three distinct regions have been described that support populations of lung tissue stem cells: intercartellagenous regions of tracheobronchial airways [6], neuroepithelial bodies (NEB) in bronchioles [8], and the bronchoalveolar duct junction (BADJ) [7,8]. Each region harbors putative tissue stem cells that share the common properties of a relatively undifferentiated phenotype, infrequent proliferation (demonstrated through use of DNA label retention assays), and a differentiation potential that is appropriate for the compartment in which they reside. A combination of immunophenotypic and cell ablative strategies has been employed to demonstrate that bronchiolar stem cells residing within both the NEB and BADJ microenvironments represent a Clara cell secretory protein (CCSP)-expressing variant Clara cell, and that the BADJ-associated population is dual-positive for both CCSP and pro-surfactant protein C. Recent studies by Kim and colleagues suggest that CCSP/SP-C dual-positive cells can be enriched based upon their unique cell surface phenotype (Sca1^+^, CD34^+^, CD31^-^, CD45^-^) and maintained long-term *in vitro *[9]. The report by Summer *et al. *[10] adds to our understanding of the lung SP and its function by phenotypically characterizing these cells. The authors have demonstrated that there are both CD45^+ ^and CD45^- ^lung SP cells and that both of these populations are contributed to by the bone marrow compartment, as demonstrated by whole bone marrow transplantation experiments.

### Rationale for the long term assay

In a previous study, the gp91phox-/- mouse had a spontaneous pro-inflammatory phenotype and then a more exaggerated emphysematous phenotype in a cigarette smoke-induced mouse model, while a double knockout (DKO) mouse lacking in both gp91phox and MMP12 failed to develop emphysema, even after chronic cigarette smoke exposure for over a month. This model is chiefly by macrophage-driven pathogenesis, while an allergen-induced asthma model is Th2 cytokine-driven and a lymphocyte-orchestrated model. The aim of the study was to explore whether reactive oxygen species and matrix proteases may have a role in an allergic set-up where phagocytic cells are responsible for much of the downstream regulation of the inflammatory process. Further to the allergic injury model, which is mainly inflammatory in nature, we wished to explore the status of regeneration in the mouse lung and thus tease out the lung stem cell niches to reveal themselves. Hence an injury model of bleomycin-induced idiopathic pulmonary fibrosis was used.

### Rationale for the short term assay

Stem cells are known to efflux Hoechst dye very slowly, and so when flow is sorted at 90 minutes post-Hoechst incubation, the cells that efflux the dye are gated and sorted as the SP cells that are assumed to be the lung stem cells. Previously most SP studies were performed on bone marrow-derived cells. To our knowledge, there is no publication to date about sorting and *ex vivo *culture of SP cells from the lung. Ours is the first study of this nature before and seven days after a single intratracheal (i.t.) instillation of 0.075 U/ml bleomycin, the lungs from C57Bl/6 mice of three genotypes, namely wildtype (WT), gp91phox-/- (NOX-/-) and MMP12-gp91phox DKO, were digested with dispase 1.2 U/ml in 37°C for an hour and then incubated with 1:200 from a Hoechst stain. They were then aliquoted and frozen at -20°C in a 37°C water bath for 90 minutes, and flow was sorted on FACSAria gating in a small (0.1%) cell population forming the characteristic 'shoulder' of actively dye-effluxing cells. These cells were then plated in mouse embryonic stem (ES) medium^+ ^in straws in liquid nitrogen. The mice were grouped as WS mice (WT + saline) and WB mice (WT + bleomycin) and were characterized by surface marker expression through 5 days of culture and then frozen.

The studies presented here were to approach a fundamental question in pulmonary pathophysiology; that is, what are the mechanisms that dictate tissue repair after both acute and chronic injury? This is a question that is central to our understanding of how tissue repair takes place under certain pathophysiological conditions and not under others. We have used knockout mice that differ in their fibrotic response to bleomycin, to tease out differences in cellular populations that may be responsible for the induction of tissue injury or repair in the short (14 days) and long term (10 weeks).

## Materials and methods

### Mice

Both gp91phox-/- mice [11] (Jackson Laboratories, Bar Harbor, ME, USA) and mmp12-/- mice were on a C57Bl/6J background and had been outcrossed and then intercrossed for three generations to generate animals deficient in both genes. C57BL6 mice (Taconic) (WT) were used as the control group. In total the following number of animals used in each group were as follows: (1) WT group-control (or with alum only), 14; ovalbumin (OVA)-treated, 16; (2) NOX-/- group-control, 14, OVA-treated, 15; (3) MMP12NOX-/- group-control, 16; OVA-treated, 14. Animals were maintained under strict specific pathogen-free (SPF) conditions following guidelines set down by the University of Washington Institutional Animal Care and Use Committee (IACUC) under protocol numbers 21-6404 and 2437-05.

### Mouse model of bleomycin-induced pulmonary fibrosis

A single i.t. dose of 0.075 U/ml of bleomycin in 40 μl saline was administered (day 0), and mice were sacrificed 14 and 21 days later. The C57Bl/6 mice were kept under animal bio-safety level (ABSL)-2 conditions approved by the IACUC of the University of Washington, and were monitored daily. They were housed under SPF conditions and were given food and water *ad libitum*. They were sacrificed on day 14. One week after bleomycin administration, mice developed marked interstitial and alveolar fibrosis, detected in lung sections by Masson's trichrome stain. Analysis of cell populations by enzymatic digestion by collagenase type IV was followed by cell counting in a Z1 Beckman Coulter particle counter (Bechman Coulter Inc, CA, USA), and subsets were identified and quantified by Flow Cytometry (FCM), and total and differential count of hematoxylin and eosin (H&E)-stained cytospin smears of single cell suspensions show loss of type I and type II alveolar epithelial cells and influx of macrophages. Alveolar epithelial cells types I and II (AEI and II) were isolated following standard protocol.

### Bromodeoxyuridine pulse chase

Bromodeoxyuridine (BrdU) is a DNA analogue. Slow cycling cells are assumed to be stem cells and pulsing of control vs. bleomycin-treated, with a single i.t. dose of 0.075 U/ml bleomycin given to WT C57Bl/6 mice at 12 hr intervals over two, four and six days intraperitoneal (IP), and pulse chase over ten weeks was expected to yield BrdU^+ ^and BrdU^- ^cells. While negative cells are assumed to be mature regularly cycling cells, BrdU^+ ^cells after ten weeks of chase are most likely label-retaining cells (LRC), that is, slow cycling stem cells that start cycling late and hence retain the label for longer. C57Bl/6 mice were given the i.t. instillation of bleomycin under brief isofluorane anesthesia, and animals were maintained under SPF conditions in the University of Washington (UW) animal facilities and sacrificed periodically to assess the above.

### Institutional approval

All animal experimentation methodology was approved by the University of Washington IACUC, protocol numbers 21-6404 (WRH) and 2437-05 (TP).

### Bronchoalveolar lavage

After pulmonary function testing, mice underwent exsanguination by intra-orbital arterial bleeding and then bronchoalveolar lavage (BAL) (0.4 ml three times) of both lungs. Total BAL fluid cells were counted from a 50 μl aliquot and the remaining fluid was centifuged at 200 *g *for 10 minutes at 4°C, and the supernatants were stored at -70^°^C for assay of BAL cytokines later. The cell pellets were re-suspended in FCS and smears were made on glass slides. After air drying, the cells were stained with Wright-Giemsa (Biochemical Sciences Inc., Swedesboro, NJ, USA) and their differential count was taken under a light microscope at 40 × magnification. Cell number refers to that obtained from lavage of both lungs in each mouse.

### Lung parenchyma

Lung mincing and digestion was performed after lavage as described previously [11] with 100 u/ml collagenase for 1 hr at 37°C, and filtered through a 60# sieve (Sigma, Aldrish Co, St Louis, MO, USA). All numbers presented in this paper refer to cells obtained from one lung/mouse. The cells recovered were primarily from the lung parenchyma (LP), as the lungs were thoroughly exsanguinated after ligating the vena cava to stop drainage into the pulmonary circulation, prior to lavage and enzymatic separation of parenchyma cells.

### Lung histology

Lungs of other animals of the same group were fixed overnight at 4°C in 4% paraformaldehyde. The tissues were embedded in paraffin and cut into 5 μm sections. A minimum of 15 fields was examined by light microscopy. The intensity of cellular infiltration around pulmonary blood vessels was assessed by H&E staining. Airway mucus was identified by staining with alcian blue and periodic acid Schiff staining as described previously [12].

### Fluorescin-activated cell sorter (FACS) analysis

Cells from hemolysed peripheral blood (PB), bone marrow (BM), BAL, LP, spleen, mesenteric lymph nodes (MLN), cervical lymph nodes (CLN), axillary lymph nodes (LNX) and inguinal lymph nodes (LNI) were analyzed on a FACSCalibur (BD Immunocytometry Systems, San Jose, CA, USA) using the CELLQuest program. Staining was performed using antibodies conjugated to fluorescin isothiocyanate (FITC), phycoerythrin (PE), allophucocyanin (APC), peridinin chlorophyll protein (Per CP-Cy5.5) and Cy-chrome (PE-Cy5 and PE-Cy7). The following BD Pharmingen (San Diego, CA, USA) antibodies were used for cell surface staining: APC-conjugated CD45 (30F-11); FITC-conjugated CD3 (145-2C11); PE-Cy5-conjugated CD4 (RM4-5);PE-conjugated CD45RC (DNL-1.9); APC-conjugated CD8 (53-6.7); PE-Cy5 conjugated B220 (RA3-6B2); FITC-conjugated immunoglobulin (IgM); PE-conjugated CD19 (ID3); PE-conjugated CD21 (7G6); FITC-conjugated CD23 (B3B4); APC-conjugated GR-1 (RB6-8C5) and PE-conjugated Mac1 (M1/70). PE-Cy5 conjugated F4/80 (Cl:A3-1(F4/80)) was obtained from Serotec Ltd., Oxford, UK. PE-conjugated anti-α4 integrin (PS2) and anti-vascular cell adhesion molecule (VCAM)-1(M/K-2) were obtained from Southern Biotechnology, Birmingham, AL, USA. Irrelevant isotype-matched antibodies were used as controls. For the non-hematopoietic cells of the lung, the following markers were from Santa Cruz, CA, USA, were used as unlabelled primary antibodies: surfactant protein (SP)-C; Oct-3/4; stage-specific embryonic antigens (SSEA)-3 and 4; thyroid transcription factor (TTF)-1; aquaporin (AQP)-1 and AQP-5. An FITC-labeled secondary antibody was used for FACs detection. Thus, all the FACS surface marker expression experiments were single marker analysis, not combinatorial. We took 10^6 ^cells per sample in 50 ml cell suspension in ice-cold PBS (16) and 10^5 ^events were recorded per sort [13]. To eliminate differences in the expression of activation markers by the choice of enzymes used for isolation, a comparative was done with collagenase, trypsin and dispase. Of these, dispase was found to be the least modulating of marker expression, and digestion by this resulted in higher BrdU^- ^cells and less SP, showing that somehow cells were effluxing dyes at a higher rate when digested thus. At this stage we can only speculate as to the effect of these enzymes on marker expression [14].

### CFU-c assay

To quantify committed progenitors of all lineages, colony-forming unit in culture **(**CFU-C) assays were performed using methylcellulose semisolid media (Stemgenix, Amherst, NY, USA) supplemented with an additional 50 ng of stem cell factor (Peprotech, Rocky Hill, NJ, USA) per ml. Next, 50,000 cells from BM, 500,000 cells from spleen, 0.01 million cells from lung and BAL fluid (BALf), and 10 μl PB were plated on duplicate 35-mm culture dishes and then incubated at 37°C in a 5% CO_2_-95% air mixture in a humidified chamber for seven days. Colonies generated by that time were counted using a dissecting microscope, and all colony types (that is, burst-forming units-erythroid (BFU-e), CFU-granulocyte-macrophage (CFU-GM), and CFU-mixed (CFU-GEMM)) were pooled and reported as total CFU-C. Total CFU-c per organ was calculated by extrapolating CFU-c against number of plated cells to the total number of cells in the organ.

### Statistical analysis

Statistical differences among samples were tested by Student *t- *test. A *P *value less than 0.05 was considered statistically significant.

## Results

### Bleomycin-induced idiopathic pulmonary fibrosis model

**Figure 1:**
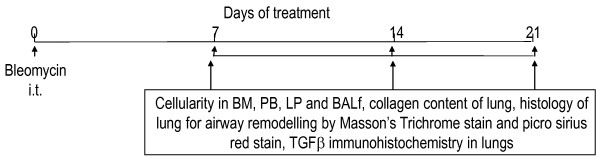
**Study design to generate fibrosis in mouse lung**. C57Bl/6 female mice (8 to 10 weeks of age) were intra-tracheally instilled with 0.075 U/ml bleomycin in 40 ml volume under brief isofluorane anesthesia, and animals were maintained under SPF conditions in the University of Washington animal facilities, and sacrificed periodically to assess the above. Abbreviations: i.p., intra-peritoneal; i.t., intra-tracheal; BALf, bronchoalveolar lavage fluid; PB, peripheral blood; TGFβ, transforming growth factor beta.

C57Bl/6 female mice (eight to ten weeks of age), instilled with bleomycin, were maintained under SPF conditions in the UW animal facilities and sacrificed periodically to the parameters shown in Figure 1. Lung fibrosis, ancillary inflammatory parameters and inflammatory cell recruitment patterns were studied.

#### Assessment of extent of fibrotic and inflammatory damage in the lung post bleomycin treatment

**Figure 2:**
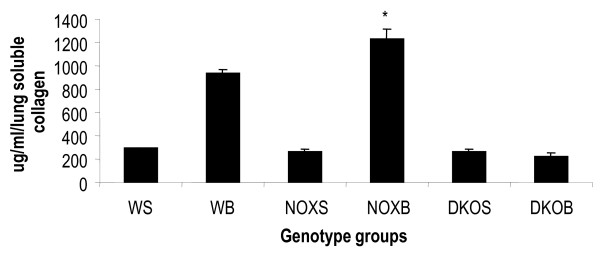
**Increase in collagen content of lung post-bleomycin in gp91phox-/- but not in MM12-gp91phox double knockout mice**. Total collagen content freshly synthesized in lung was measured by Sircol assay. Data represent mean of 2 independent experiments, *n *= 4/group. Data are expressed as avergae ± SEM. *P < 0.05 compared to the post-OVA value in wildtype mice. Abbreviations: WS, saline-treated wildtype (baseline); WB, bleomycin-treated (single intra-tracheal dose of 0.075 U/ml bleomycin and animals were sacrificed 14 and 21 d later. Here day 21 data are shown); NOXS, saline-treated gp91phox null; NOXB, bleomycin-treated gp91phox null; DKOS, saline treated MMP12- gp91phox double knockout; DKOB, bleomycin-treated MMP12- gp91phox double knockout.

Mice were sacrificed at 10 weeks and blood, LP enzymatically digested by dispase 1.2 U/ml and BALf (lavage volume 400 μl × 3 with ice cold PBS) were analyzed. The total number of cells was assessed by the Coulter particle counter. PB was extrapolated to 2 ml (equal to the volume of total PB in a 20 gm mouse), LP of both lungs and BALf were also analyzed for both lungs in each mouse (Figure 2).

**Figure 3:**
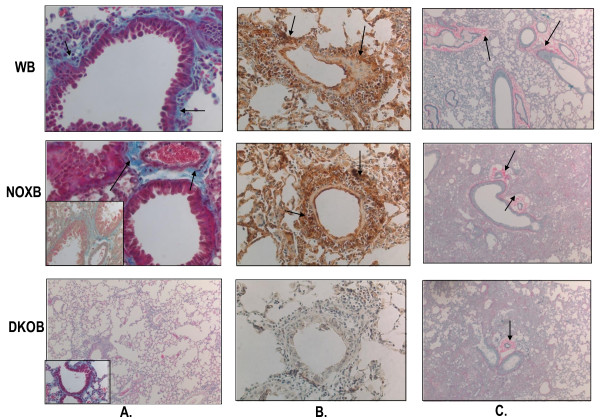
**Increased inflammation and fibrosis in lung of WT and gp91phox-/- mice but not MMP12gp91phox double knockout mice**. At 21d post-bleomycin treatment, mice were sacrificed and lungs were fixed overnight in 4% paraformaldehyde and embedded in paraffin. Sections of 8 m were dye-stained by **A**, Masson's Trichrome stain and **C**, Picro Sirius red and were immunostained with TGFb antibody and color developed by Diaminobenzidine (DAB). (**B**). Arrows indicate blue collagen deposits (**A)**, TGFb positive stain (**B**) and red collagen staining (**C**). In **A**, pink-purple stains indicate cytoplasm, and blue the nuclei of inflammatory cells. Light blue stains are collagenous deposits. While wildtype and gp91phox-/- (designated as NOX-/-) show increased collagen deposits and inflammation around airways post-ovalbumin (all sections are those of post- ovalbumin lung), the MMP-12-gp91phox double knockout lung shows minimum collagen deposition as well as TGFb expression. While **A **and **B **were at 60 × magnification, **C **was at 10 × magnification. **A **inset shows another area of collagen deposition in bleomycin-treated wildtype; NOXB and DKOB were bleomycin-treated gp01 phox-/- and double knockout mice respectively. The photomicrographs were taken using an Olympus VX4L microscope and Nikon digital camera. All photomicrographs were at 60 × magnification. Abbreviations: WB, wildtype mice treated with bleomycin; NOXB, bleomycin-treated gp91phox null; DKOB, bleomycin-treated MMP12- gp91phox double knockout.

Total freshly synthesized tropocollagen quantified in the post-bleomycin lung of WT and gp91phox-/- mice show respectively a 3-fold and 4.6-fold increase compared to their untreated or placebo-treated counterparts. DKO mice strikingly showed no increase in collagen content and lung homogenate showed tropocollagen at similar levels to that of untreated mice (Figure 2). By histology and histochemical and immunohistochemical staining, LP and areas surrounding small bronchoalveolar ducts show enhanced inflammatory recruitment, increased transforming growth factor (TGF)-β secretion, and collagen deposits around small and large airways (Figure 3).

#### Inflammatory cell accumulation in the lungs and airways over time post-bleomycin treatment

Table 1 shows significant inflammation in the lungs and airways by day 7 after bleomycin treatment. While WT mice had a 7-fold increase in total inflammatory cell number in the BALf compared to untreated WT mice post-bleomycin, gp91phox-/- had more than 10-fold increase, but DKO had no appreciable inflammation in the untreated or treated lung. Inflammatory cell components in the airways of bleomycin-treated lungs in WT mice were mostly lymphocytes (157-fold increase) and neutrophils (150-fold increase) while macrophages increased 2.6-fold. In the gp91phox-/- mice, however, there was a slight shift in the inflammatory cell recruitment profile, while in the post-bleomycin lung, lymphocytes (187-fold increase) occupied a higher share of the inflammatory exudates in proportion to the other cell types, which shows apportionment of a 6-fold higher number of macrophages, and a 35-fold increase in the number of neutrophils compared to the control. Therefore, in the gp91phox-/- lung the shift is in the myeloid population, which showed a somewhat diminished migration to airways compared to the lymphoid population, although overall inflammation was more pronounced than in the WT mice. In DKO mice, the airways contained a slightly higher number of inflammatory cells post-bleomycin, but the number was similar to the WT control and was not significantly enhanced.

**Table 1:** Number of hematopoietic and non-hematopoietic cells in BALf and lung a week after bleomycin treatment - trends in cellular migration to BALf and lung post-bleomycin over time

A. Number of cells in BALF after a week of bleomycin treatment								
	**WT saline (day 7)**		**WT bleomycin (day 7)**	**NOX saline (d7)**	**NOX bleomycin (day 7)**	**DKO saline (day 7)**		**DKO bleomycin (day 7)**

**Total cell count (×10^5^/ml)**	4.27 ± 0.184		28.3 ± 0.224	4.515 ± 0.223	48.91* ± 1.59	2.61 ± 0.16		8.42 ± 0.13

**Macrophages (×10^5^/ml)**	4.16 ± 0.57 (97.2%)		11.14 ± 4.08 (37.6%)	4.01 ± 0.31 (89.9%)	28.14* ± 6.83 (57.47%)	1.87 ± 0.64 (71.64%)		4.19 ± 3.18 (49.82%)

**Lymphocytes (×10^5^/ml)**	0.08 ± 0.01 (2.0%)		12.63 ± 2.49 (46.0%)	0.09 ± 0.02 (2.01%)	16.91* ± 3.65 (34.53%)	1.2 ± 0.01 (4.59%)		1.44 ± 0.66 (17.12%)

**Neutrophils (×10^5^/ml)**	0.03 ± 0.01 (0.7%)		4.5 ± 0.97 (16.3%)	0.11 ± 0.01 (0.02%)	3.91 ± 0.53 (7.98%)	0.63 ± 0.14 (24.13%)		2.78 ± 1.06 (33%)

**B. Number of CD45^+ ^cells in BALf before and after Bleomycin treatment**								

**BALf**								

**10^5^/ml**	**WS**	**WB7**	**WB14**	**WB21**	**NOXS**	**NOXB21**	**DKOS**	**DKOB21**

**Total cells**	4.27 ± 0.18	28.3 ± 0.22	10.32 ± 2.74	9.32 ± 1.74	4.51 ± 0.23	8.36 ± 1.32	4.55 ± 0.16	5.03 ± 1.94

**Macrophage**	4.16 ± 0.57	11.14 ± 4.08	8.91 ± 4.93	7.06 ± 3.92	3.86 ± 1.07	6.91 ± 1.96	3.91 ± 0.74	4.03 ± 0.43

**Lymphocyte**	0.08 ± 0.01	12.63 ± 2.49	1.76 ± 0.67	1.33 ± 0.67	1.32 ± 0.54	2.43 ± 0.43	1.44 ± 0.32	0.56 ± 0.21

**PMN**	0.03 ± 0.01	4.5 ± 0.97	0.41 ± 0.07	0.87 ± 0.32	0.67 ± 0.32	1.02 ± 0.22	0.67 ± 0.11	0.41 ± 0.13

**C. Number of (CD45+) cells in lung parenchyma before and after bleomycin treatment**								

**LP (d7)**								

**10^5^/m1**	**WS**		**WB**	**NOXS**	**NOXB**	**DKOS**		**DKOB**

**Total cells**	7.27 ± 0.07		38.3 ± 4.04	8.86 ± 0.08	54.19* ± 2.6	6.85 ± 0.27		9.56 ± 0.65

**Macrophage**	6.16 ± 0.57		22.14 ± 4.08	7.13 ± 2.43	31.38* ± 11.32	5.41 ± 1.43		6.36 ± 2.71

**Lymphocyte**	1.08 ± 0.01		9.63 ± 2.49	0.97 ± 0.12	21.41* ± 4.93	0.93 ± 0.21		2.47 ± 0.67

**PMN**	0.03 ± 0.01		7.5 ± 0.97	0.72 ± 0.04	1.34 ± 0.15	0.51 ± 0.03		0.73 ± 0.33

**%**	**WS**		**WB**	**NOXS**	**NOXB**	**DKOS**		**DKOB**

**Macrophage**	84.73 ± 1.86		57.80 ± 11.75	80.47 ± 12.96	57.96 ± 2.98	78.97 ± 3.76		66.52 ± 5.98

**Lymphocyte**	14.85 ± 2.84		25.14 ± 4.96	10.94 ± 1.95	39.50 ± 3.67	13.57 ± 3.98		25.83 ± 8.43

**PMN**	0.41 ± 0.04		19.58 ± 6.73	8.12 ± 2.07	2.47 ± 1.09	7.44 ± 1.97		7.63 ± 2.06

**D. Number of CD45- cells in lunb parenchyma counterstained with alveolar epithelial cell markers before and after bleomycin treatment**								

	**saline**			**bleomycin**				

**WT**	**Day 21**			**Day 7**	**Day 14**	**Day 21**		

**AEI**	95.7 ± 4.87			79.47 ± 2.86	67.43 ± 1.76	58.41 ± 4.83		

**AEII**	4.3 ± 1.76			9.41 ± 1.86	8.66 ± 1.07	5.96 ± 1.12		

**NOX-/-**								

**AEI**	95.7 ± 3.87			66.21 ±	58.23 ± 2.34	56.14 ± 3.97		

**AEII**	4.3 ± 1.12			4.89 ±	5.43 ± 1.06	5.77 ± 0.56		

**DKO**								

**AEI**	95.7 ± 4.16			96.23 ± 4.75	96.44 ± 11.23	97.41 ± 3.87		

**AEII**	4.3 ± 0.56			4.5 ± 0.09	4.86 ± 1.94	4.96 ± 1.87		

**A**. Number of cells in BALf after a week of bleomycin treatment. **B. N**umber of CD45^+ ^cells in BALf before and on days 7, 14 and 21 after bleomycin treatment (one intratracheal dose of 0.074 U/ml in 40 ml volume). **C**. Number of (CD45^+^) cells in lung parenchyma (LP) before and after bleomycin treatment. **D**. Number of CD45- cells in LP counterstained with alveolar epithelial cell markers before and after bleomycin treatment. After the single intra-tracheal dose of bleomycin, animals were sacrificed on days 7, 14 and 21 for the WT after bleomycin, and at days 7 and 21 only for the NOX-/- and DKO mice and WS. Cell number was quantitated by hemocytometer and corroborated by Z1 particle counter (Beckman Coulter). Number of cells represent data from 2 independent experiments ± SEM. *n *= 4/group. Cell subsets were quantified by FACS and morphologic differentiation under light microscope. Olympus BX41 at 40 × magnification. CD45+ cells were gated and macrophages (Gr1-F4/80hi), lymphocytes (CD3^+ ^and B220^+^), and neutrophils (Gr-1hi F4/80^-^) were identified, their percentages analyzed by CellQuestPro and their total numbers calculated from the total number of cells obtained by lavage as described in materials and methods. Data presented were pooled from 2 independent experiments ± SEM. *P < 0.05 compared to WT post-bleomycin on corresponding time points). Abbreviations: WT (wild type), BALf, bronchoalveolar lavage fluid; NOX-/- (gp91phox knockout), gp91phox MMp12 double knockout (DKO); WS, WT + saline (day 7); WB7, WT + post-bleomycin day 7; WB14, WT + post-bleomycin day 14; WB21, WT + post-bleomycin day 21; NOXS, gp91phox-/- + saline day 21; NOXB21, gp91phox-/- + bleomycin day 21; DKOS, gp91phox-MMP12 double knockout + saline (day 21), DKOB21, gp91phox-MMP12 double knockout + bleomycin (day 21).

### Stem cell niches characterization in the bleomycin-induced injury model

#### Long term BrdU pulse chase assay

**Figure 4:**
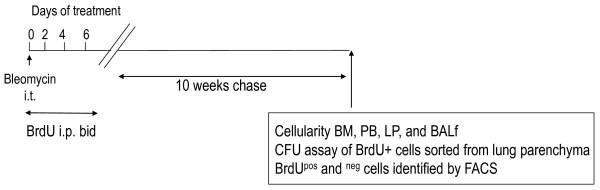
**Study design of BrdU pulse chase of control and bleomycin-induced lung fibrosis model in mice**. C57Bl/6 mice had intratracheal instillation of 0.075 U/ml bleomycin in 40 ml volume under brief isofluorane anesthesia and animals were maintained under SPF conditions in the University of Washington animal facilities, and sacrificed periodically to assess the above. Slow cycling cells are assumed to be stem cells and pulsing of control vs. bleomycin-treated wildtype C57Bl/6 mice over two, four and six days i.p. at 12-hr intervals and pulse chase over 10 weeks, was expected to yield BrdU^+ ^cells and BrdU^- ^cells. While negative cells are assumed to be mature regularly cycling cells, BrdU^+ ^cells after 10 weeks of chase are most likely label retaining cells (slow cycling stem cells) that started cycling late and hence retain the label for longer. BrdU is a DNA analogue. Abbreviations: i.p. intra-peritoneal; i.t. intra-tracheal; BALf, bronchoalveolar lavage fluid; BM, bone marrow; PB, peripheral blood. BrdU, bromodeoxyuridine; FACS, fluorescin-activated cell sorter.

**Figure 5:**
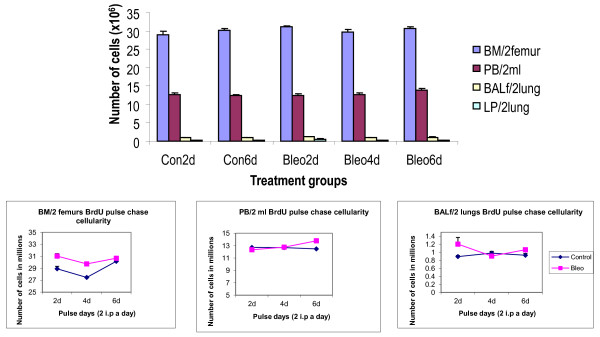
**Cellularity in bone marrow, peripheral blood, lung parenchma and bronchoalveolar lavage fluid before and after bleomycin after 10 weeks of bromodeoxyuridine pulse chase**. Total cell number in bone marrow, blood, lung and airways after 10 weeks of chase following12-hourly pulsing for up to 6 days in the saline-treated control mice (Con) and bleomycin-treated (Bleomycin) (one i.t. dose of 0.075 U/ml). *P *values < 0.05 compared to the control were considered significant. Data were averaged from three independent experiments. FACsans and flow sorts were done in triplicate per sample. Samples were counted in duplicate tubes per sample for each data point (*n *= four to five per group). Mortality was less than 2%. In this experiment no significant change was found in total cellularity. Abbreviations: BM, bone marrow; PB, peripheral blood; BALf, bronchoalveolar lavage fluid; LP, lung parenchyma; BrdU, bromodeoxyuridine.

A long-term assay was performed to detect stem cell niches exposed by the bleomycin-induced injury model (Figure 4) [13]. Total cellularity was measured (Figure 5). Marker expression analysis by FACS was used to help identify BrdU^+ ^cells (possibly stem cells) and BrdU^- ^(either accessory cells characterized by their typical surface marker expression or parenchyma other than stem cells) were assessed as percent total cells by FCM by conjugated monoclonal antibodies (Table 2). The total number of cells remained unaltered in BM, PB, lung and BALf. Probably after 10 weeks of chase, whatever temporary alteration in cells had occurred in the short term (one to three weeks post-bleomycin), reached equilibrium at the end of ten weeks. BrdU^+ ^cells are the stem cells, and are identified by the marker expression on them, thus, double-positive cells are stem cells and are characterized by their marker expression. BrdU^- ^cells are the remaining cells that are also characterized by their marker expression. These are presumably the non-stem cells that may be accessory cells if present adjacent to the stem cell niches (to be corroborated by Immunohistochemistry (IHC) by spatial distribution. The percentages shown in the datasheet were obtained by gating on BrdU^+ ^or BrdU^- ^cells.

**Table 2:** Percent positive cells by FACS within BrdU^+ ^and BrdU^- ^populations in lung for pluripotent and pulmonary lineage-specific markers

A. Percent positive cells by FACS within BrdU^+ ^population in lung									
	**BrdU+**	**TTF-1+**	**Oct3/4+**	**SSEA-3+**	**SSEA-4+**	**Sca-1+**	**Lin-**	**CD34+**	**CD31+**

**Con 2d**	8.51 ± 0.23	20.55 ± 0.30	86.18 ± 1.05	40.9 ± 0.83	48.85 ± 0.25	932 ± 1.70	95.54 ± 0.78	1.08 ± 0.02	2.39 ± 0.10

**Con 6d**	9.05 ± 0.03	21.11 ± 0.59	85.67 ± 0.93	42.90 ± 1.11	46.44 ± 2.24	87.66 ± 2.56	92.30 ± 0.66	1.58 ± 0.20	1.92 ± 0.63

**Bleo 2d**	2.67 ± 0.12	4.16 ± 0.12	78.25 ± 2.01	8.91 ± 0.02	8.97 ± 0.025	3.67 ± 0.09	44.82 ± 1.75	2.42 ± 0.17	4.77 ± 0.18

**Bleo 4d**	1.83 ± 0.21	4.52 ± 0.45	80.58 ± 0.12	8.07 ± 0.15	8.57 ± 0.41	3.71 ± 0.30	38.05 ± 1.54	3.01 ± 0.49	4.60 ± 0.19

**Bleo 6d**	1.87 ± 0.07	4.37 ± 0.29	79.62 ± 0.74	8.12 ± 0.53	7.95 ± 0.043	4.46 ± 0.24	36.28 ± 1.79	3.32 ± 0.40	3.60 ± 0.26

	**SP-C+**		**AQP-5**	**CC-10**	**CD45+**	**CD45-**	**SP-C+** **CC10+**		**VEGF+**

**Con 2d**	4.69 ± 0.13		0.83 ± 0.02	2.52 ± 0.15	2.13 ± 0.02	96.93 ± 0.90	82.80 ± 1.53		15.67 ± 0.86

**Con 6d**	4.59 ± 0.26		0.8 ± 0.004	2.42 ± 0.24	2.53 ± 0.23	93.95 ± 2.34	81.08 ± 0.42		13.78 ± 0.42

**Bleo 2d**	0.23 ± 0.05		0.14 ± 0.01	2.67 ± 0.09	3.42 ± 0.12	91.79 ± 1.60	2.43 ± 0.08		8.59 ± 0.29

**Bleo 4d**	0.54 ± 0.08		0.14 ± 0.02	3.16 ± 0.05	3.5 ± 0.20	90.38 ± 0.77	2.4 ± 0.56		8.70 ± 0.28

**Bleo 6d**	0.32 ± 0.04		0.14 ± 0.009	3.29 ± 0.12	3.57 ± 0.21	87.25 ± 2.29	2.08 ± 0.18		7.78 ±

**B. Percent positive cells by FACS within BrdU^- ^population in lung**									

	**BrdU-**	**TTF-** **1+**	**Oct3/** **4+**	**SSEA** **-3+**	**SSEA** **-4+**	**Sca-** **1+**	**Lin-**	**CD3** **4+**	**CD3** **1+**

**Con 2d**	92.13 ± 0.84	0.38 ± 0.10	0.560 ± 0.09	0.55 ± 0.02	0.47 ± 0.14	0.22 ± 0.15	0.2 ± 0.01	0.31 ± 0.06	0.03 ± 0.01

**Con 6d**	92.95 ± 0.71	0.31 ± 0.07	0.35 ± 0.02	0.4 ± 0.07	0.32 ± 0.03	0.06 ± 0.02	0.19 ± 0.04	0.22 ± 0.009	0.22 ± 0.09

**Bleo 2d**	89.06 ± 0.66	11.36 ± 0.59	1.11 ± 0.09	0.28 ± 0.07	0.35 ± 0.02	0.12 ± 0.07	0.34 ± 0.03	3.45 ± 0.09	0.71 ± 0.042

**Bleo 4d**	89.43 ± 0.66	12.13 ± 0. 74	1.34 ± 0.09	0.32 ± 0.04	0.26 ± 0.08	0.25 ± 0.03	0.24 ± 0.08	3.42 ± 0.23	0.8 ± 0.09

**Bleo 6d**	97.40 ± 1.1	11.68 ± 0.6	1.742 ± 0.16	0.31 ± 0.04	0.32 ± 0.01	0.16 ± 0.05	0.54 ± 0.05	2.90 ± 0.06	0.6 ± 0.086

	**SP-C+**		**AQP-5+**	**CC-10**	**CD45+**	**CD45-**	**SP-C+** **CC10+**		**VEGF+**

**Con 2d**	5.59 ± 0.11		90.92 ± 0.50	1.30 ± 0.18	2.37 ± 0.17	80.38 ± 1.36	0.03 ± 0.01		2.86 ± 0.09

**Con 6d**	4.90 ± 0.14		85.75 ± 2.21	1.33 ± 0.21	2.7 ± 0.16	83.70 ± 1.66	0.12 ± 0.03		2.31 ± 0.14

**Bleo 2d**	1.48 ± 0.17		9.29 ± 0.41	2.60 ± 0.16	0.78 ± 0.06	76.15 ± 1.69	0.01 ± 0.004		12.74 ± 0.59

**Bleo 4d**	1.74 ± 0.03		217.49 ± 208.5	2.85 ± 0.08	0.65 ± 0.13	74.65 ± 1.37	0.02 ± 0.004		12.45 ± 0.84

**Bleo 6d**	1.52 ± 0.06		9.05 ± 0.18	2.36 ± 0.19	0.81 ± 0.13	74.05 ± 1.65	0.12 ± 0.06		10.65 ± 0.26

Bromodeoxyuridine-positive (BrdU^+^) cells were gated and inside (positive, and outside of the gate, BrdU-negative) cells were characterized by FACS with fluorochrome conjugated monoclonal antibodies markers of pluripotence (TTF-1, SSEA-3,4, Oct ¾, SCA-1, lineage negative) and CD31 (endothelial progenitor) and CD34 (hematopoietic progenitor). Lung lineages are characterized by the following markers: AQP-5^+ ^cells are AEI, SP-C^+ ^cells are AEII and CC-10^+ ^cells are Clara cells while SP-c^+^CC-10^+ ^cells are bronchoalveolar stem cells. The gating strategies were as follows: BrdU^+ ^and BrdU^- ^cells were first gated and then the other markers were detected within the gates. Gating was first done on BrdU^+ ^and BrdU^- ^cells and then individual markers such as TTF-1, Oct3/4, SSEA 3, 4, SCA-1^+^, Lin-, CD34^+ ^and CD31^+ ^cells were assessed. The percentages, therefore, do not add up to 100% as only single positive cells are separately assessed in each gate. It is possible that there may be some redundancy but overall, it is possible have a rough idea about the kind of cells that are label-retaining cells and therefore the putative stem cells within the mouse lung. Con, control group; Bleo, bleomycin-treated group.

#### Detection of stem cells by CFU-C

**Figure 6:**
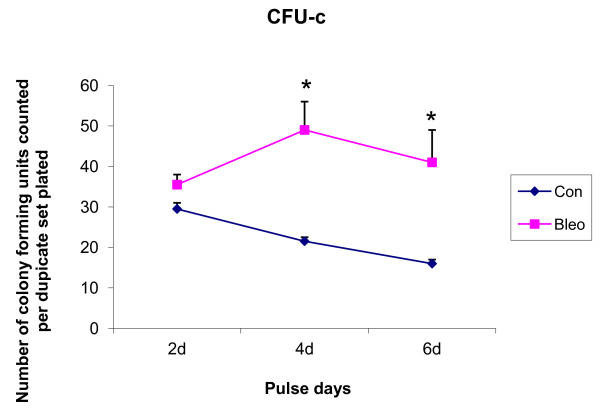
**Colony forming units in lung from bromodeoxyuridine-positive cells sorted after 10 weeks of chase**. Bromodeoxyuridine single-positive cells in the lung parenchyma were decreased 4.8-fold post-bleomycin, thus, the putative stem cell population appeared gradually depleted from lung upon bleomycin treatment. The 0.1 × 10^6 ^cells were plated in semi-solid methyl cellulose and CFU were counted after 14 days. All three data points (pink squares) for bleomycin-treated (Bleo) mouse lungs were significantly increased compared to the control (Con) mouse (blue diamonds). *P < 0.05 was considered statistically significant. Data were pooled from three independent experiments (*n *= 4 or 5 per group). Mortality was less than 2%. Averaged data are presented as mean ± SEM of three experiments. Data were collected in duplicate petri dishes for each sample.

BrdU single^+ ^cells in the lung parenchyma were decreased 4.8-fold post-bleomycin. Putative stem cell populations seemed to be gradually depleted from the lung upon bleomycin treatment. We plated 0.1 × 10^6 ^cells in semi-solid methyl cellulose and performed CFU-counts after 14 days (Figure 6). BrdU^+ ^cells from mouse lungs collected from bleomycin-treated mice on day 2, day 4 and day 6 of the pulse chase were statistically significantly increased.

### Detection of stem cells by marker expression

#### Characterization of BrdU^+ ^cells

Only BrdU^+ ^cells were sorted and plated, as these are the slow cycling stem cells derived from the lung. Marker expression was studied by FACS analysis to characterize stem and mature differentiated cells (Table 2). TTF-1, which is a typical expression marker of AEII, has been implicated in early lung development. Post-bleomycin, there was a 4.9-fold decrease in these cells, showing that putative progenitors are destroyed. Oct3/4 is an early pluripotent marker. Post-bleomycin there was a one-fold decrease in the percent Oct3/4^+ ^cells among BrdU+ cells. This may indicate a universal downregulation of pluripotency post-bleomycin and may be due to an overall depletion of the stem cell reserve, or individual cells may lose Oct3/4 expression in limited pockets (calculation of the kd value by Scatchard analysis may give a more exhaustive quantitative assessment).

Post-bleomycin, SSEA-3 and 4 were decreased 5.9- and 6.14-fold, respectively, compared to the control. Since these are stem cell-specific antigens, this may again point towards an overall depletion of pluripotent reserve cells in niches. Sca-1^+ ^cells are mouse-specific stem cells. Being BrdU^+^, these were undoubtedly stem cells in the lung. A 22.6-fold decrease in Sca-1-BrdU double^+ ^cells may also indicate that the post-bleomycin progenitor population decreased in the lung. Lineage-negative cells are those that are non-hematopoietic in origin and here, being BrDU^+^, are the definitively non-hematopoietic cells in lung that have stem-like properties. A 2.6-fold decrease in these cells in bleomycin-treated mouse lungs indicates that stem cells of non-hematopoietic origin were depleted. CD34^+ ^cells are hematopoietic cells and being BrdU^+^, must also have stem-like properties. A 2.2-fold increase in these cells post-bleomycin indicates that hematopoietic stem cells definitely had an impact post-bleomycin on lung stem cell content. Our theory is that either hematopoietic stem cells from bone marrow or other tissues may travel to injured lung to replenish the depleted lung stem cell niche.

CD31^+ ^cells are endothelial progenitors. There was a 1.9-fold increase in these cells, which are also BrdU^+^. SP-C^+ ^cells are AEII cells. There was a 14.3-fold decrease post-bleomycin. AEII are established progenitors that are assumed to trans-differentiate to AEI upon bleomycin-induced depletion of the former. Being BrdU^+ ^here, they are conclusively slow cycling cells, although expressing SP-C, a marker of mature AEII cells. This is interesting because decrease in this double-positive population shows either that pre-destined SP-C^+ ^LRC are depleted post-bleomycin or that SP-C^+ ^cells, which revoke their progenitor property and become slow cycling, probably as a preamble to trans-differentiation into AEI, become increasingly depleted. The percentage of AQP-5^+ ^AEI cells was quite low to start with and became lower post-bleomycin. However, as a technical note, since the percentage of positive cells was extremely low to start with (control), the 6-fold decrease post-bleomycin may not be significant.

Some Clara cells have also been traditionally known to possess stem-like properties (for instance, naphthalene resistance). Here CC-10^+ ^BrdU^+ ^cells increased by 1.2-fold post-bleomycin, showing that Clara cells probably have some definite responsive function to a bleomycin challenge.

#### Characterization of non-stem cells (BrdU-)

These are the normally cycling cells, presumably the other cells constituting the rest of the LP. There was no significant difference between the pre- and post-bleomycin BrdU^- ^populations in the mouse lung. BrdU^+ ^and BrdU^- ^cells showed the expected relative distribution in the same scattergram making 100%. TTF-1^+ ^BrdU^- ^cells were decreased 36.78-fold. Since TTF-1 is co-expressed on AEII, increase in this cell population may indicate an alteration in AEII response to bleomycin. Whether this has any significance to stem cell niches in the lung is unknown. Oct3/4+BrdU- cells were decreased by 4.97-fold post-bleomycin. Therefore, along with the stem cell population, the mature cells were also probably universally depleted to be replaced by collagen fiber.

The percentage of SSEA-3, SSEA-4 and Sca-1^+ ^cells did not differ before and after bleomycin when gated on BrdU^- ^cells. These markers were expressed on an extremely low number of cells and hence may be of no consequence in this response. Lineage-negative cells showed a statistically significant 2.8-fold increase. Again this may indicate that non-hematopoietic cells in the lung are universally depleted post-bleomycin. Again the percentage of this population was so low that this may be technically ignored. CD34^+ ^hematopoietic progenitors that were not LRC were also concommitantly increased 14.5-fold post-bleomycin, again corroborating our hypothesis that there is a recruitment of progenitors of hematopoietic origin in response to bleomycin. Endothelial progenitors CD31^+ ^cells were also increased 2.9-fold post-bleomycin. These are the non-stem CD31^+ ^in the lung. We do not know how this may be significant. However, again the cell percent was extremely low.

SP-C+ AEII were decreased 3.2-fold and AQP-5^+ ^AEI were decreased 9.5-fold. This is in keeping with the overall concept of degeneration of alveolar epithelium in bleomycin-induced fibrosis. Clara cells were decreased by 1.3-fold and this was statistically significant. CD45^+ ^and CD45^- ^cells were decreased 3.3-fold and 1.1-fold post-bleomycin, again in keeping with the overall degeneration of lung cell parenchyma in bleomycin-induced fibrosis.

### Short term Hoechst SP experiment

#### Analysis of inflammatory cells post-bleomycin treatment in single and double knockout mice

**Figure 7:**
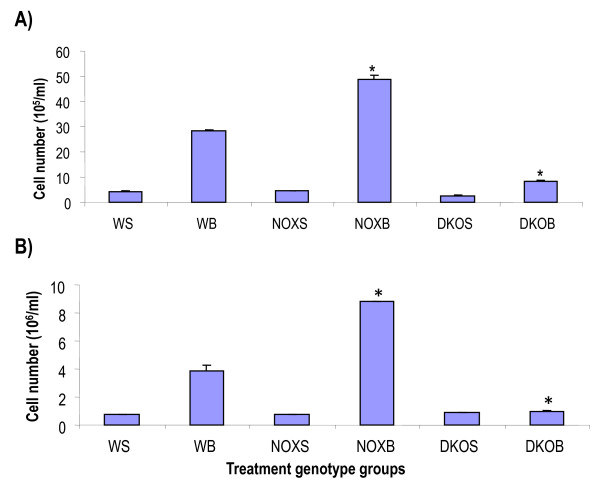
**Cellularity in bronchoalveolar lavage fluid and lung before and after bleomycin challenge at day 7 in wildtype vs. gp91phox-/- and MMP12-gp91phox double knockout mice**. Airways were lavaged and cells counted by a Z1 particle counter from Beckman Coulter. Lung lobes were excised after thorough exsanguination of the mouse by infra-orbital bleeding after ligation of the superior vena cava. Minced parenchyma was digested with dispase and the resultant cell suspension, filtered of cell debris, was stained with Hoechst dye, and cellularity assessed. *P < 0.05 compared to control, averaged from three independent experiments. FACsans and flow sorts were done in triplicate per sample. Samples were counted in duplicate tubes per sample for each data point (*n *= 4 to 5 per group). Mortality was less than 2%. Abbreviations: WS, saline-treated wildtype mice; WB, wildtype mice treated with bleomycin; NOXS, saline-treated gp91phox null; NOXB, bleomycin-treated gp91phox null; DKOS, saline treated MMP12- gp91phox double knockout; DKOB, bleomycin-treated MMP12- gp91phox double knockout.

Cellularity of the LP was measured in single cell suspension by Z1 Beckman particle counter. Only LP post-bleomycin (day 7) were considered, as our aim was to look for stem cells in the lung, and the changes in cell number are more apparent at day 7 than by day 21. The cell number of BALf and lung did not show any significant difference between groups before and after bleomycin treatment, and the histopathologic assessment of the lung showed conclusive proof of the fibrotic process having occurred in the lung (Figure 7).

#### SP cells isolated from pre- and post-bleomycin treatment (day 7)

**Figure 8:**
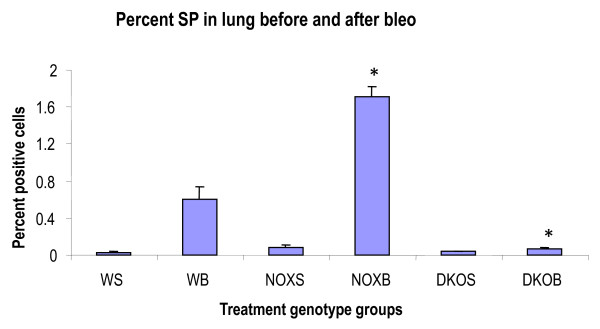
**Percent side population cells in lung before and seven days after bleomycin challenge in wildtype vs. gp91phox-/- and MMP12-gp91phox double knockout mice**. At this point, lung parenchyma was digested by dispase and Hoechst dye effluxing cells (side population) were isolated. *P < 0.05 compared to control, averaged from three independent experiments. FACsans and flow sorts were done in triplicate per sample. Abbreviations: WS, saline-treated wildtype mice; WB, wildtype mice treated with bleomycin; NOXS, saline-treated gp91phox null; NOXB, bleomycin-treated gp91phox null; DKOS, saline treated MMP12- gp91phox double knockout; DKOB, bleomycin-treated MMP12- gp91phox double knockout.

Marker expression by FACS was used when next we sought to characterize the cells thus sorted (Figure 8), and was isolated and cultured *ex vivo *to note the changes in marker expression over time. Our future plan with these cells is to differentiate them into lung lineage-specific cells and monitor their functional efficacy in regenerating lung tissue in bleomycin-induced fibrosis. For characterization, each mouse group of four mice lungs were pooled and cell culture was sampled with detached cell suspension (0.05% trypsin-EDTA) for FCM. So, day 1 to day 5 refers to the days during which these cells that were sorted from day 7 post-bleomycin mouse lungs, were cultured in mouse ES medium. The samples, each pooled from lungs of four mice (1-4) at 7 days post-bleomycin, will be termed WS 1-4, (WT + saline), WB 1-4 (WT+bleomycin), NOXS1-4 (gp91phox-/-+saline), NOXB1-4 (gp91phox-/-+bleomycin), DKOS1-4 (MMP12-gp91phox double knockout+saline), and DKOB1-4 (MMP12-gp91phox double knockout+bleomycin).

### Cells in BALf and lung post-bleomycin over time

The total number of cells and cell subsets was measured periodically over time in WT and knockout mice (Table 1). The total number of cells was increased in bleomycin-treated NOX-/- mice at day 7 after bleomycin, with macrophages and lymphocytes contributing most of the increase. The percentage of Polymorphonuclear neutrophils (PMNs) also increased but the total number was comparable to that in post-bleomycin WT mice. DKO mice, on the other hand, had a slightly higher total number of cells than the saline-treated control group, with lymphocytes and PMNs contributing entirely to the slight increase. Compared to saline-treated WT mice, the number of cells increased 6.6-fold one week after bleomycin treatment. This was denuded at day 14 and further denuded at day 21. Therefore, similar to previous data, cell number in BALf was not an indicator of the extent of fibrosis by day 21. Similar trends were found in the lung (Table 1C). Day 7 probably signals the onset of fibrosis by increased cellular recruitment. Macrophages and T cells are the chief secretors of TGFb, traditionally thought to activate collagen synthesis and deposition by alveolar epithelial cells. Day 8 to day 21 therefore, is the scar tissue formation period when AEI and AEII have become denuded. This is shown in Table 1D (Figure S1 in Additional file 1), where there is a progressive decrease in AEI through day 21 while AEII first increased slightly only to equilibriate at day 21. The NOX-/- BALf showed a 1.7-fold increase in cell number, which decreased predictably by day 21. DKO BALf, however, showed no appreciable increase over saline-treated mice, either at day 7 or at day 21. Macrophages seem to be the chief cell populations accounting for this increase. In lung (Table 1C) (Figure S2 in Additional file 1) however, on day 7, a similar trend to that in BALf is found. The only difference is that both macrophages and lymphocytes make up for the increase.

### Characterization of sorted SP cells in *ex vivo *culture

**Figure 9:**
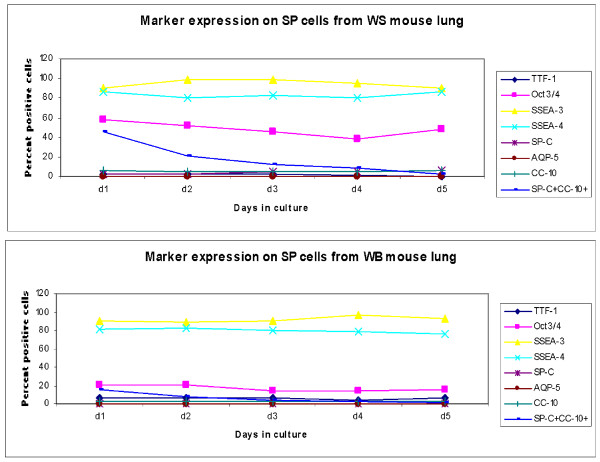
**Marker expression on wildtype side population cells flow sorted and cultured for five days**. Lung parenchyma, homogenized and digested with dispase, was incubated with Hoechst dye for 90 minutes in a water bath at 37°C. After washing three times with cold PBS, cells were flow sorted with the FACS ARIA cell sorter from Becton Dickinson. The sorted side population (SP) cells, gated from the typical arch formed in the scattergram by Hoechst positive cells, were kept in *ex vivo *culture in ES medium with LIF and assessed for markers by FCM every day for five days. Percent positive cells for markers on SP cells sorted and cultured in mouse ES medium with LIF. FACsans and flow sorts were done in triplicate per sample. *P < 0.05 compared to control, averaged from three independent experiments. Abbreviations: WS, wildtype mice treated with saline; WB, wildtype mice treated with bleomycin; d1-5, days 1 to 5 in culture.

**Figure 10:**
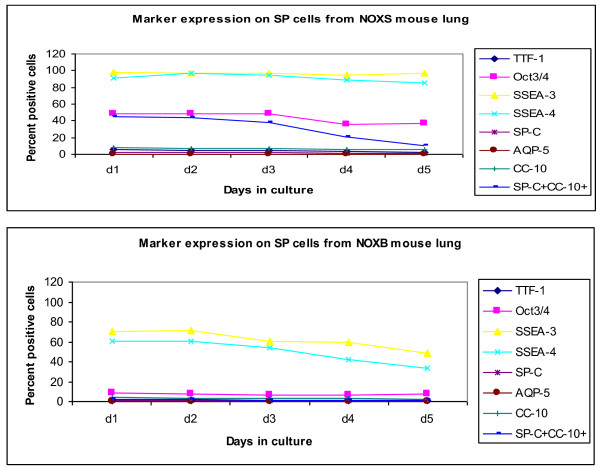
**Marker expression on NOX-/- side population cells flow sorted and cultured for five days**. Lung parenchyma, homogenized and digested with dispase, was incubated with Hoechst dye for 90 minutes in a water bath at 37°C. After washing three times with cold PBS, cells were flow sorted with the FACS ARIA cell sorter from Becton Dickinson. The sorted side population (SP), cells gated from the typical arch formed in the scattergram by Hoechst positive cells, were kept in *ex vivo *culture in ES medium with LIF and assessed for markers by FCM every day for 5 days. Percent positive cells for markers on SP cells sorted and cultured in mouse ES medium with LIF. FACsans and flow sorts were done in triplicate per sample. *P < 0.05 compared to control, averaged from three independent experiments. Abbreviations: WS, wildtype mice treated with saline; WB, wildtype mice treated with bleomycin d7; d1-5, days 1 to 5 in culture; NOXS, saline-treated gp91phox null; NOXB, bleomycin-treated gp91phox null.

**Figure 11:**
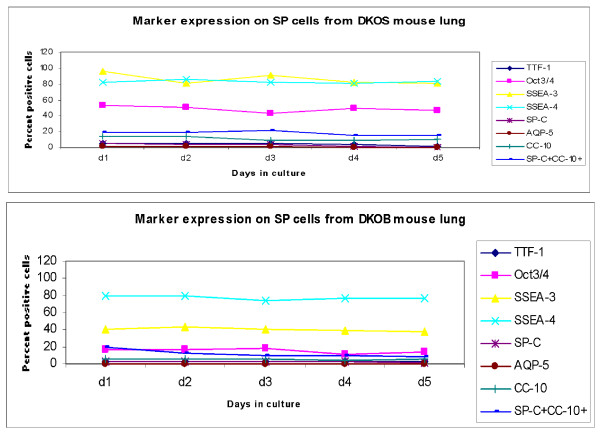
**Marker expression on DKO SP cells flow sorted and cultured for 5 days**. Lungs parenchyma, homogenized and digested with dispase, was incubated with Hoechst dye for 90 mins in a water bath at 37°C. After washing thrice with cold PBS, cells were flow sorted with FACS ARIA cell sorter from Becton Dickinson. The sorted Side Population cells gated from the typical arch formed in the scattergram by Hoechst positive cells were kept in *ex vivo *culture in ES medium with LIF and assessed for markers by FCM every day for 5 days. Percent positive cells for markers on SP cells sorted and cultured in mouse ES medium with LIF. FACsans and flow sorts were done in triplicate per sample. *P < 0.05 compared to control, averaged from three independent experiments. Abbreviations: WS, wildtype mice treated with saline; WB, wildtype mice treated with bleomycin d7; d1-5, days 1 to 5 in culture; DKOS, saline treated MMP12- gp91phox double knockout; DKOB, bleomycin-treated MMP12- gp91phox double knockout.

Sorted cells from day 7 post-bleomycin mice (Figure 8) and their corresponding control groups from all three genotype groups were plated in mouse ES, and FACScan of surface expression markers was evaluated in culture every 24 h, before freezing on day 5 of culture. Thus the data represent the percentage positive for each marker expression on flow-sorted SP cells in *ex vivo *culture with Leukemia Inhibitory Factor (LIF) (Figures 9, 10, 11). The total number of cells that could be expanded was small, to the tune of 10^4^. Most of the cells were therefore stained and scanned. Cell death was low, as viability count was taken before staining, and > 90% cells were alive (data by Trypan blue dye exclusion is not presented). The stains were again done separately and scattergrams were collected in un-gated cells and monostained cells (so they do not add up to 100%) except for SP-C and CC-10, which were done together, especially to identify double positive cells, which are thought to be the putative stem cells.

In the bleomycin-induced fibrosis experiments performed earlier and repeated with this latest batch of mice, it is apparent that while gp91phox-/- mice respond with an exaggerated fibrotic manifestation in lung, MMP12- gp91phox DKO mice show little or no difference from their saline-treated counterparts. Isolation of SP cells from the lungs of these animals on day 7 post-bleomycin and *ex vivo *culture for five days may explain the mechanism for such response on the basis of changes in their marker expression in culture with LIF (to not promote differentiation).

Overall, SSEA-3 and 4 remained stationary at quite high expression throughout the culture period, with both untreated lung cells from all three genotype groups as well as bleomycin-treated WT lung cells, except for the two knockout mice post-bleomycin, where both were significantly downregulated. Other pluripotent marker expression was downregulated in all three post-bleomycin, but of note is the different expression of SP-C-CC-10 double-positive cell population in both saline-treated and bleomycin-treated knockout cells. The significance of this difference is unknown.

## Discussion

It is generally important to elucidate airway epithelial cell lineages and to identify multipotent progenitors as targets for gene therapy. Stem cells are typically present in specialized compartments spatially proximal to their differentiated progeny, but an equivalent paradigm has not been demonstrated in the airway. We discovered a distinct population of cells displaying high levels of keratin expression in murine tracheal submucosal gland ducts, and tested the hypothesis that BrdU LRCs, thought to represent stem cells, were present in this compartment. Mice received weekly epithelial damage by intratracheal detergent or SO_2 _inhalation for 4 wk and received intraperitoneal injections of BrdU every 48 h during the injury and repair period. At three and six days after injury, BrdU-positive epithelial cells were noted along the entire tracheal length in both basal and lumenal cell positions. At later time points (20 and 95 days) LRCs were localized to gland ducts in the upper trachea, and to systematically arrayed foci in the lower trachea, typically near the cartilage-intercartilage junction. LRCs were not pulmonary neuroendocrine cells. Heterotopic tracheal grafts after surface epithelial removal demonstrated reconstitution of a surface-like epithelium from gland remnants.

On the other hand, SP cells are believed to be derived from the bone marrow and can be differentiated from committed tissue stem cells. In models of ablative radiation injury, CD45^+ ^lung SP cells have demonstrated transient repopulation of the bone marrow and indicated that CD45^+ ^lung SP cells are analogous to the short-term repopulating hematopoietic cell. In models of ablative radiation injury, CD45^+ ^SP cells have been demonstrated to be sufficient for reconstitution of the bone marrow [15,16]. In addition, in one report, marked SP cells were shown to repopulate damaged lung [17] in irradiated mice, resulting in rare, but detectable, fibroblasts and alveolar and bronchial epithelial cells. These findings support the position that tissue SP cells are hematopoietically derived, pluripotent stem cells that may play an important role in tissue repair. Other studies have attempted to address whether SP cells that are localized to specific tissues maintain their pluripotency. Distinct populations of tissue-localized SP or Lin^-^, Sca-1^+ ^cells that maintain hematopoietic activity have been identified in muscle [18,19] and liver [18]. Interestingly, these SP cells have not been identified in PB and are least frequently found in the bone marrow (0.79% of nucleated cells) compared with other tissues (0.96 to 15.1% of nucleated cells) [20]. These results suggest that airway epithelial stem cells are localized to specific niches [21].

The bleomycin-induced idiopathic pulmonary fibrosis model in WT and knockout mice was developed in a systematic study to investigate whether inflammation may be involved down- or upstream of the onset of the fibrotic process as well as to tease out the lung stem cell niches by inflicting injury to the tissue (Figure 1). In other words, the degeneration of functional AEI and AEII and other pulmonary cell types help reveal stem cell niches by the sheer force of mobilization of putative progenitors that herald the onset of the regeneration of lost tissue. Identification of the stem-like characteristics of the mobilized or *homing *progenitors' long term assays (Figures 4, 5, 6, Table 2) and short term assays (Figures 7, 8, 9) revealed that post-bleomycin treatment there was heavy inflammation in the lung parenchyma as well as the airways (Figures 2, 3, Table 1). The presence of a large number of myeloid inflammatory cells recruited to the airways and obtained in the exudates, shows this inflammation to be mainly Th1-driven.

As communicated in our publication [22], both inflammation and airway hyperreactivity were more extensive post-OVA in NOX or gp91phox knockout mice than in WT mice. Although OVA-specific IgE in plasma were comparable in WT and knockout mice, enhanced inflammatory cell recruitment from circulation and cytokine release in lung and BALf, accompanied by higher airway resistance, as well as enhanced pause in response to methacholine, indicate a regulatory role for nicotinamide adenine dinucleotide phosphate (NADPH) oxidase in the development of allergic asthma. Also, while T cell-mediated functions like Th2 cytokine secretion, and proliferation to OVA were upregulated synchronous with the overall robustness of the asthma phenotype, macrophage upregulation in functions such as proliferation, mixed lymphocyte reaction, and MCP-1-directed chemotaxis, but downregulation of respiratory burst response, indicate a forking in their signaling pathways. The gp91^*phox*^-/- MMP12 DKO mice show a similar phenotype to the gp91^*phox*^-/-, showing the non-involvement or synergistic involvement of MMP12 in the response pathway. Contrary to a Th2-driven inflammation as in this OVA-induced allergic asthma model, bleomycin-induced IPF.Idiopathic Pulmonary Fibrosis (IPF) obviously takes a different afferent immune response pathway to develop. However, the most striking finding is with the fibrosis model, where there is no inflammation in the DKO mice, as well as no fibrosis, indicating that inflammation is a prerequisite to fibrosis.

The BrdU^+^CD45^+ ^population comprises hematopoietic cells with pluripotent properties. Post-bleomycin they are increased 0.7 fold, again corroborating our theory that progenitors from circulation form a component of the lung stem cell scenario. Most BrdU^+ ^cells were CD45^- ^cells (non-hematopoietic origin). There was no significant alteration in their number when assessed by gating on CD45^- ^alone. SP-C^+^CC-10^+ ^cells are the broncho-alveolar stem cells (BASCs). Gated BrdU^+ ^cells show a 38.9-fold decrease in this population, which formed the majority of the BrdU^+ ^cells before, and constituted a bare 2% post-bleomycin in the mouse lung. This is perhaps the most important finding of the pulse chase study, that BASCs are so drastically mobilized and depleted after 10 weeks of bleomycin-induced fibrosis. VEGF is a growth factor traditionally associated with increased vascularization. Since fibrosis is a degenerative disease, we measured VEGF^+ ^cells in the BrdU^+ ^population and found that there was a 1.8-fold decrease post-bleomycin. If VEGF is assumed to be a pro-fibrotic signal, then decrease in VEGF+ LRC may indicate either that the fibrotic process is slowing wearing off, or VEGF is assumed to be a signal for progenitor mobilization. Of significance is that there is no change in the BrdU^- ^BASC population. Does this mean that there is a very small quiescent population of these cells, even though the there is a BrdU^+ ^BASc, which is otherwise responsive to a bleomycin challenge? VEGF^+ ^cells were increased 4.6-fold post -bleomycin. Interestingly, while the VEGF^+ ^LRC were decreased, VEGF^+ ^BrdU^- ^cells were increased post-bleomycin. This may help counterbalance the mobilization of VEGF^+ ^cells from stem cell niche (Table 2A) (Figure S3 in Additional file 1). There was no significant difference in the colony-forming potential of BrdU^- ^cells before and after bleomycin after the 10 week chase.

SP cells were flow-sorted from lungs of mice at seven days post-bleomycin exposure. This was done primarily on the assumption that the fibrosis process was just beginning. However, as apparent from cell traffic data in the airways (Table 1A-C), especially the real-time trend on days 7, 14 and 21, speak of a primary inflammatory recruitment followed by an exodus out of the lung that is slowly replaced by fibrosis. This was significant. SP cells from day 7, therefore, are the progenitors that are mobilized coinciding with the inflammatory cell mobilization from circulation. It may be that inflammatory cells produce signals to induce mobilization of lung stem cells from tissue niche or they may bring with them progenitors mobilized from the bone marrow. Whatever the origin, lineage commitment may be occurring when the same cells cultured *in vitro *show up- or downregulation of different markers.

Fibrosis induced by bleomycin was a deliberate injury given to the lung tissue to tease out progenitors residing within the tissue for exigencies. In the process, the study aimed to identify, isolate and characterize stem cells of the adult lung. Hence we used the bleomycin-induced pulmonary fibrosis model. It is true that multiple progenitors exist in the lung, as the reviewer has suggested. However, the scope of the present study was simply to characterize the cells with stem-like properties rather than enumerate all the different kinds that exist there, the ramification of which is beyond the scope of this study. We have addressed colony-forming units in the injured vs. healthy lung and shown that both LRC and SP increase, and we additionally identified CD34^+ ^and CD31^+^, putative hematopoietic and endothelial progenitors within the tissue (Table 2).

Fibrosis sets in when functional epithelial, muscular and endothelial cells making up the structural architecture of the lung are progressively degenerated and replaced by extra cellular matrix protein (ECM). The injury axis of SDF-1α-CXCR4 typically regulates mobilization and homing of pluripotent or limited lineage-committed hematopoietic and non-hematopoietic cells from bone marrow, peripheral circulation, secondary lymphoid tissue and lung tissue itself, to try to replenish damaged tissue and reverse fibrosis. The perturberance of normal physiological homeostasis of the lung recalls cells to replace dead or damaged cells, mostly AEII in the lung [22].

At the beginning of their life outside the lungs (Figures 9, 10, 11), the numbers of sorted SP cells expressing mature cell markers were very low but as the culture progressed in the presence of LIF (under negative selection pressure where differentiation is inhibited) markers of stem-like characteristics were downregulated, indicating that despite the presence of LIF, a slow maturation process has begun. Of note is the expression of the Oct3/4 pluripotent marker, which was about 16% (*P *< 0.05) lower than in the NOX compared to saline-treated WT as well as saline-treated DKO mice. This unexpected down-modulation may account for the unregulated development of fibrosis in the single knockout (gp91phox-/-) mouse only, as opposed to the DKO mouse, which is protected. Whether the MMP12 deletion in addition to the NADPH oxidase sub-unit deletion has a counteractive and therefore protective effect towards development of fibrosis is speculative as it is beyond the scope of this paper, and needs more work with the Wnt/β catenin pathway to reveal molecular mechanisms underlying this protection.

On the other hand, cellular senescence has been shown to be a state of irreversible growth arrest [23], characterized by distinct morphological and biochemical changes and induced either by telomere shortening or by telomere-independent signals such as DNA damage and oxidative stress; using AEII DNA-damaging agents, such as bleomycin, induced a senescence phenotype. Regeneration of the injured epithelium, which involves proliferation of AEII cells, was seriously affected, resulting in the absence of appropriate re-epithelialisation. This pathway of impaired epithelial cell regeneration, leading to pulmonary fibrosis, suggests that proliferation of AEII is important for preventing the progress of bleomycin-induced pulmonary fibrosis. The senescent cells produced higher levels of matrix metalloproteinases, such as collagenases and stromelysin, and of profibrotic cytokines, such as interleukin (IL)-1β and TGF-β, and indicates that senescent cells may be involved not only in impaired tissue regeneration, but also in the inflammatory response and extracellular matrix remodelling.

The extent of decrease in its expression in the bleomycin-treated cells was also 2-fold greater in the gp91phox-/- compared to WT and DKO mice-derived cells. The other interesting modulation in marker expression is that of the double-positive BASC cells (SP-C^+^CC-10^+^) that was sharply downregulated by day 2 of culture in the WT, but showed a much more gradual downregulation in the gp91phox-/- (d4) mice. The DKO mice on the other hand was 2-fold lower than the first two, in both the saline- and the bleomycin-treated mice, while the percentage of these BASC in the WT and gp91phox-/- mice decreased almost to nil (since this is an *ex vivo *culture excluding cell turnover per se, we may assume that the markers themselves downregulate). In the absence of more detailed study, we may simply comment that the rate and degree of BASC may have a developmental significance in the progression of fibrosis. Mesenchymal cells, as well the stroma of the lungs, may have a significant role to play in the future development of the cells [24-26].

As a commentary and hypothesis, we offer the following views: the cells within a tissue such as the lung, where rapid turnover of cells is the rule where efficient regeneration is needed to balance the high level of oxidative wear and tear, in the light of the data presented in this paper, we believe that the identity of a cell, both structural and functional, undergoes a dynamic plasticity by which it can change back and forth between functionally competent lineage-restricted cells of the different anatomical tissue sub-types of the lung and progenitor-like cells that can differentiate and trans-differentiate into cells of the required type, according to the current demand to restore homeostatsis in the lung [27]. To accomplish this there must be efficient and seamless de-differentiation and differentiation of the same cellular pool by simply switching certain defined molecular switches on and off. The data showing the dynamics of marker expression in the lung and airways of an injured lung in real time testify to this. The so-called *niches *therefore may be both anatomical, occupying distinct regions of the lung, as well as functional, that is irrespective of their spatial distribution, cells can undergo rapid changes as to their developmental identity according to the exigent situation born out of a particular injury [26]. Studies [28-31] describe how cells fished out from adult tissue using similar strategies, and suitably engineered, can be an unlimited source of cells to counter the process of degeneration. The above study provides valuable insight into this translational aim.

## Conclusion

In summary, interpreting data presented in this communication, the following conclusions may be drawn: Firstly, the long term BrdU pulse chase experiments infer that (1) the increase in the hematopoietic progenitor pool in the lung indicated that exogenous progenitors from the circulation contribute to lung regeneration; (2) most non-stem cells (possibly accessory cells) were non-hematopoietic in origin indicating that despite tissue turnover and some spontaneous resolution of fibrosis as reported, BASCs are drastically depleted, possibly necessitating recruitment of progenitors from the hematopoietic pool; (3) loss of VEGF^+ ^LRC may indicate either a gradual resolution of the fibrosis process or a signal for progenitor mobilization from niches; (4) the BrdU^- ^BASC population may be a small quiescent population that remains as a reserve for more severe lung injury; (5) increase in VEGF^+ ^non-LRC may indicate a checkpoint to counterbalance the mobilization of VEGF^+ ^cells from the stem cell niche and a protective measure against complete depletion of the same. Secondly, from the short term SP population analysis studies, the following conclusions can be drawn: (1) inflammation is a prerequisite for fibrosis; (2) SP cells, being the putative stem cells in the lungs, were increased (either by self renewal or by recruitment from the exogenous bone marrow pool) post-bleomycin in NOX-/- but not in DKO mice, indicating the necessity of cross-talk between gp91phox and MMP-12 in this process; (3) *ex vivo *cultured SP progressively lose pluripotent markers, notably BASC (SPC^+^CC10^+^) - the significance of this is unknown.

Stem/progenitor cells can be used to repair defects in the airway wall, resulting for example, from tumors, trauma, tissue reactions following long-time intubations, or diseases that are associated with epithelial damage. Several potential sources of cells for airway epithelium have been identified. These can be divided into two groups. The first group consists of endogenous progenitor cells present in the respiratory tract. This group can be subdivided according to location into (a) a ductal cell type in the submucosal glands of the proximal trachea; (b) basal cells in the intercartilaginous zones of the lower trachea and bronchi; (c) variant Clara cells (Clara v-cells) in the bronchioles; (d) variant Clara cells at the junctions between the bronchioles and the alveolar ducts and (e) alveolar type II cells. This classification of progenitor cell niches is controversial. The second group consists of exogenous stem cells derived from other tissues in the body. This second group can be subdivided into: (a) embryonic stem (ES) cells, induced pluripotent stem (iPS) cells, or amniotic fluid stem cells; (b) SP cells from bone marrow or epithelial stem cells present in bone marrow or circulation and (c) fat-derived mesenchymal cells. Airway epithelial cells can be co-cultured in a system that includes a basal lamina equivalent, extracellular factors from mesenchymal fibroblasts, and in an air-liquid interface system. Recently, spheroid-based culture systems have been developed. Several clinical applications have been suggested: cystic fibrosis, acute respiratory distress syndrome, chronic obstructive lung disease, pulmonary fibrosis, pulmonary edema, and pulmonary hypertension. Clinical applications so far are few, but include subglottic stenosis, tracheomalacia, bronchiomalacia, and emphysema [32-35].

## Abbreviations

ABSL: animal bio-safety level; APC: allophucocyanin; BADJ: bronchoalveolar duct junction; BAL: bronchoalveolar lavage; BASCs: broncho-alveolar stem cells; BM: bone marrow; BrdU: Bromodeoxyuridine; CCSP: Clara cell secretory protein; CLN: cervical lymph nodes; DAB: Diaminobenzidine; DKOB: double knockout bleomycin-treated MMP12- gp91phox double knockout; DKOS: saline treated MMP12- gp91phox double knockout; extra cellular matrix protein (ECM); ES: embryonic stem; FACS fluorescin-activated cell sorter; FITC: fluorescin isothiocyanate; hematopoietic stem cells: HSC; IgM: immunoglobulin; IL: interleukin; IP: intraperitoneal; induced pluripotent stem: Ips; IT: intratracheal; IUCAC: Institutional Animal Care and Use Committee; LNI: inguinal lymph nodes; LNX, LP: lung parenchyma; axillary lymph nodes; LRC: label-retaining cells; MLN: mesenteric lymph nodes; NEB: neuroepithelial bodies; PE: phycoerythrin; Per CP-Cy5: peridinin chlorophyll protein 5; WS: wildtype treated with saline; NOXB: bleomycin-treated gp91phox null; NOXS: saline-treated gp91phox null; OVA: ovalbumin; PB: peripheral blood; SP: side population; SPF: specific pathogen-free; TGF: transforming growth factor; WB: bleomycin-treated wildtype mice.

## Competing interests

The authors declare that they have no competing interests.

## Authors' contributions

ERB conceived and designed the experiments, analyzed the data, and wrote the paper. WRH made substantial contributions to conception and has given final approval for the version to be published.

## Supplementary Material

Additional file 1 **Figure S1 Scattergrams of CD45^- ^cells with alveolar epithelial specific markers scanned by FACS from LP of mice from different genetic groups before and after bleomycin treatment (data presented in **Table 1D). CD45^- ^cells were gated. Left panel, SP-C^+ ^AEII cells; right panel, AQP-5^+ ^AEI; AEII are WS 5.4%, WB 7.2%, NOXS 4.5%, NOXB 5.8%, DKOS 5.6%, DKOB 6.2%; AEI are: WS 93.9%, WB 60.4%, NOXS 94%, NOXB %, DKOS 90%, DKOB 92.3% in this representative scattergram of one of the three independent experiments which were averaged and presented as mean ± SEM. **Figure S2 **Scattergrams of CD45^+ ^hematopoietic cells scanned by FACS from lung parenchyma of mice from different genetic groups before and after bleomycin treatment (data presented in Table 1C). Lung parechyma (day 7). Panel 1 A-C, myeloid cells: cells are CD45^+ ^gated and macrophages and neutrophils. GR-1+^hi^F4/80+^lo ^cells are neutrophils and GR-1+^lo^F4/80+^hi ^cells are macrophages. In knockout mice, post-bleomycin lung parenchyma show a greater presence of macrophages than neutrophils, showing that these were mobilized from local niches (GR-1+^lo^). A, Macrophage (Mf)36.7%, neutrophil (PMN) 14.8%; B, Mf 56.8%, PMN 1.8% and C, Mf 59.8%, PMN 2.3%, Panel 2D-E, lymphoid cells: D, T cells; E, B cells. **Figure S3**. Scattergram of Brd^+ ^cells also individually expressing different pluripotent and pulmonary lineage specific markers (data presented in Table 2A). Wildtype mouse cells were treated with BrdU and counterstained with SP-C. The upper right (UR) quadrant shows double-positive BrdU+SP-C^+ ^cells. Click here for file
