# Supplementary material for: Characterization of lung stem cell niches in a mouse model of bleomycin-induced fibrosis
Source: Stem Cell Res Ther. 2012 May 29;3(3):21. doi: 10.1186/scrt112 (PMC3392768; doi:10.1186/scrt112)
Supplement: Additional file 1 — Figure S1 Scattergrams of CD45- cells with alveolar epithelial specific markers scanned by FACS from LP of mice from different genetic groups before and after bleomycin treatment (data presented in Table 1D). CD45- cells were gated. Left panel, SP-C+ AEII cells; right panel, AQP-5+ AEI; AEII are WS 5.4%, WB 7.2%, NOXS 4.5%, NOXB 5.8%, DKOS 5.6%, DKOB 6.2%; AEI are: WS 93.9%, WB 60.4%, NOXS 94%, NOXB %, DKOS 90%, DKOB 92.3% in this representative scattergram of one of the three independent experiments which were averaged and presented as mean ± SEM. Figure S2 Scattergrams of CD45+ hematopoietic cells scanned by FACS from lung parenchyma of mice from different genetic groups before and after bleomycin treatment (data presented in Table 1C). Lung parechyma (day 7). Panel 1 A-C, myeloid cells: cells are CD45+ gated and macrophages and neutrophils. GR-1+hiF4/80+lo cells are neutrophils and GR-1+loF4/80+hi cells are macrophages. In knockout mice, post-bleomycin lung parenchyma show a greater presence of macrophages than neutrophils, showing that these were mobilized from local niches (GR-1+lo). A, Macrophage (Mf)36.7%, neutrophil (PMN) 14.8%; B, Mf 56.8%, PMN 1.8% and C, Mf 59.8%, PMN 2.3%, Panel 2D-E, lymphoid cells: D, T cells; E, B cells. Figure S3. Scattergram of Brd+ cells also individually expressing different pluripotent and pulmonary lineage specific markers (data presented in Table 2A). Wildtype mouse cells were treated with BrdU and counterstained with SP-C. The upper right (UR) quadrant shows double-positive BrdU+SP-C+ cells. [file scrt112-S1.PPT]

## Slide 1
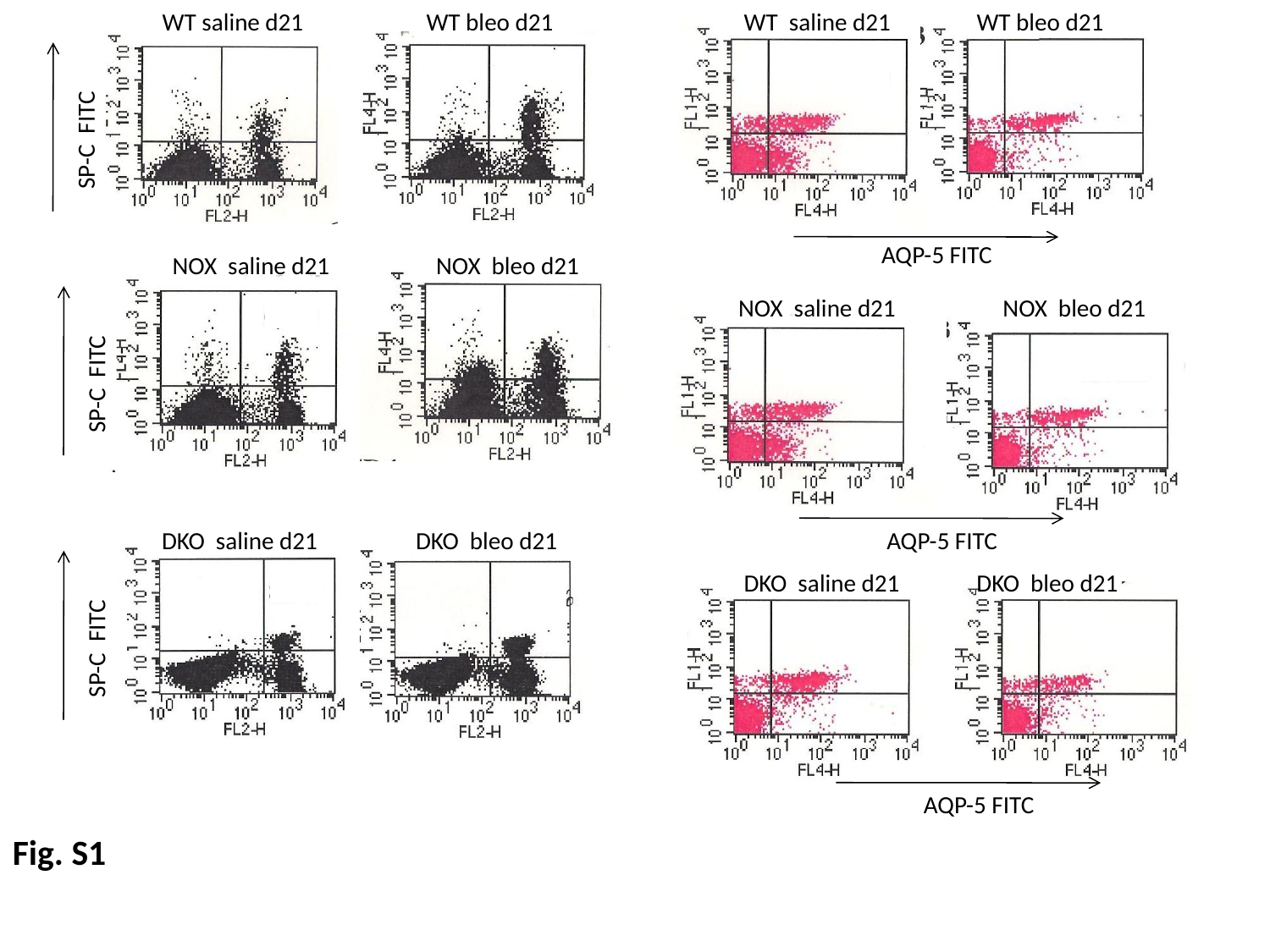

WT saline d21
WT bleo d21
SP-C FITC
WT saline d21
WT bleo d21
AQP-5 FITC
NOX saline d21
NOX bleo d21
SP-C FITC
NOX saline d21
NOX bleo d21
AQP-5 FITC
DKO saline d21
SP-C FITC
DKO bleo d21
DKO saline d21
DKO bleo d21
AQP-5 FITC
Fig. S1

## Slide 2
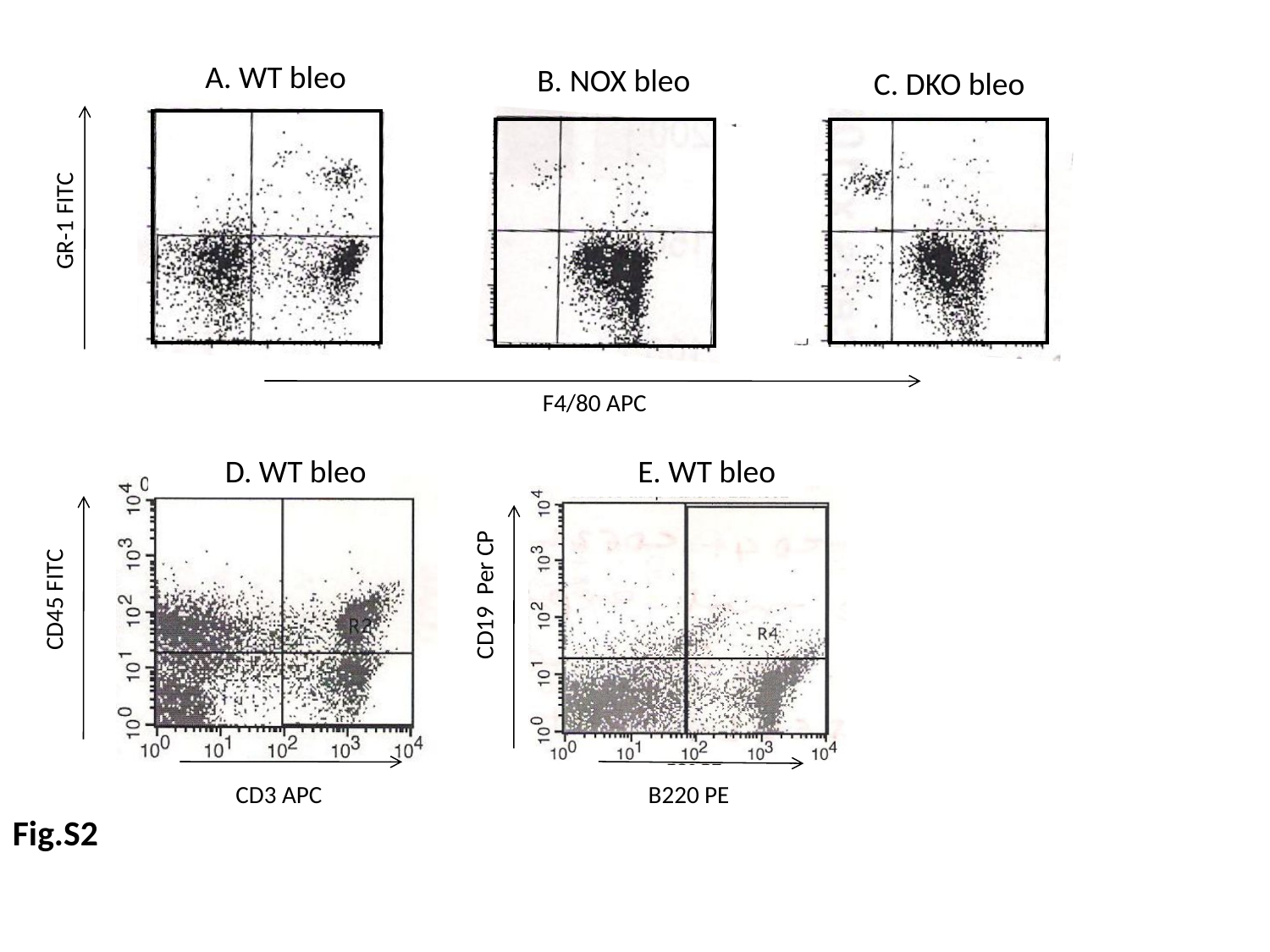

A. WT bleo
B. NOX bleo
C. DKO bleo
 GR-1 FITC
F4/80 APC
D. WT bleo
E. WT bleo
 CD45 FITC
 CD19 Per CP
CD3 APC
B220 PE
Fig.S2

## Slide 3
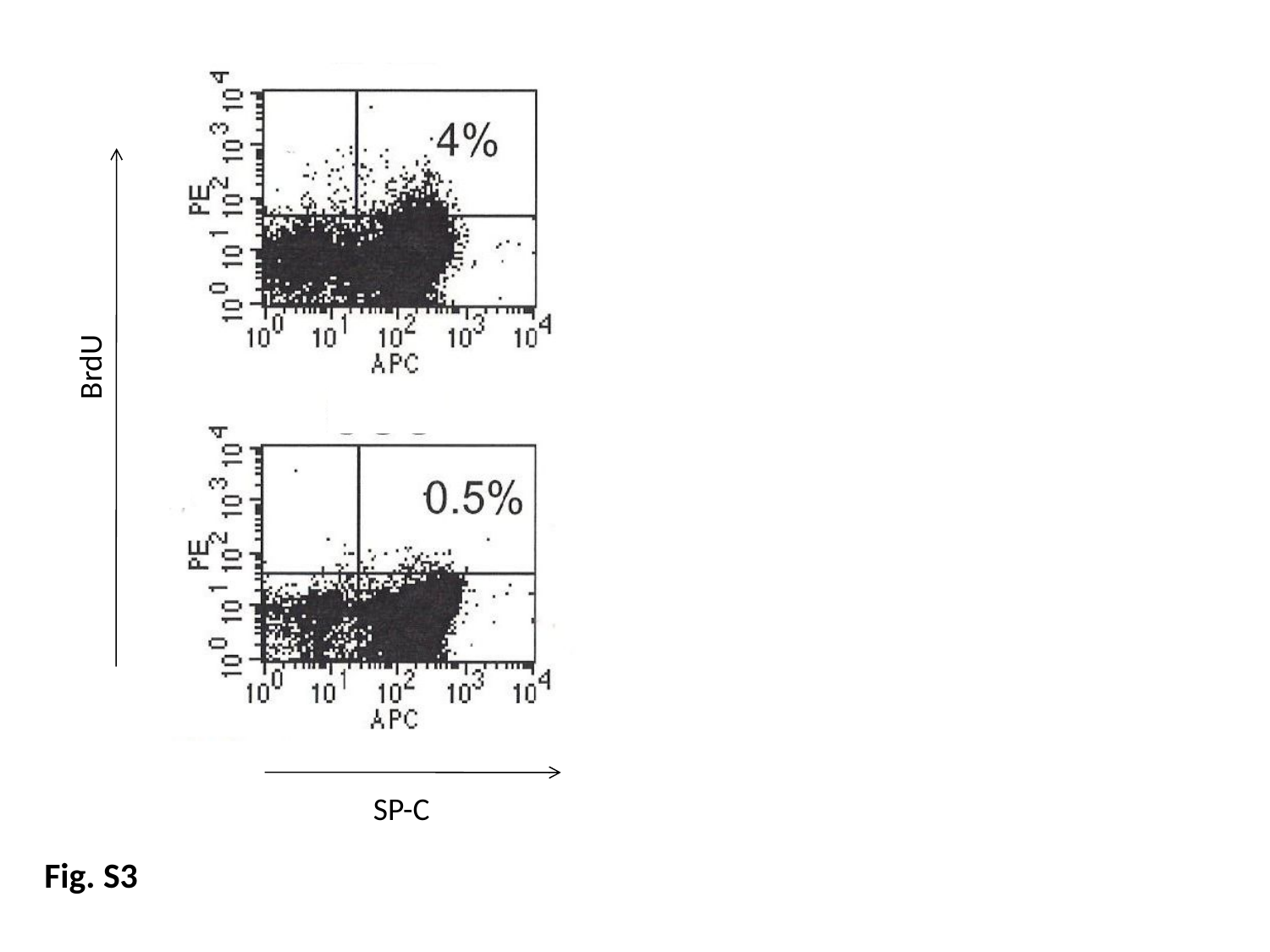

BrdU
SP-C
Fig. S3
